# Mitral valve billow and prolapse: a brief review at 45 years

**Published:** 2009-02

**Authors:** IWP Obel

**Affiliations:** Electrophysiology Laboratory, Milpark Hospital, Johannesburg

## Abstract

**Summary:**

Barlow’s syndrome has become a regular, often-used and very often misused diagnosis. Its description followed extensive, prolonged and detailed clinical observation by JB Barlow and his co-workers. This major research effort was necessary because of the protean manifestations of the condition. The differentiation of Barlow’s syndrome from other conditions with similar and sometimes identical symptoms requires clear and unambiguous criteria. These criteria were identified by penetrative clinical research. Consequently, it became possible to diagnose Barlow’s syndrome with a high degree of specificity.

Almost equally important were the gains made in understanding various conditions with similar symptoms but totally different management. An example of which, understanding some of the electrocardiographic patterns that emerge on effort in patients with ischaemic heart disease. Similarly, understanding mitral valve billow led to a greater knowledge of the entire pathophysiology of the mitral valve closure and important aspects of mitral regurgitation. Primary mitral valve billow, Barlow’s syndrome, resulted from clinical research of the highest quality and has had a major application in clinical medicine.

## Summary

In the above article, the authors emphasised that the distinctive auscultatory feature of the primary billowing mitral leaflet syndrome (BMLS) was the presence of a mitral systolic (usually late) murmur together with a non-ejection click. Associated symptoms often included chest pain, palpitations, anxiety, as well as electrocardiographic changes (resting S-T and T-wave changes), arrhythmias, conduction defects, systemic emboli and autonomic disorders.^1^ Reviewing 25 years of experience of this syndrome, the authors emphasised that a benign course is usual, requiring only reassurance. They equally emphasised that the condition may on occasion be complicated by systemic emboli, infective endocarditis, progression to severe mitral regurgitation, arrhythmias and rarely, sudden death. Importantly, Barlow and Pocock stressed that mitral valve billow and prolapse may be secondary to or associated with many conditions. The prognosis then is principally that of the underlying condition; most important of which they considered to be ischaemic heart disease and hypertrophic cardiomyopathy. Indeed, more than 32 other conditions were noted by the authors to cause or be associated with mitral valve billow (MVB) or a non-ejection click and/or mitral regurgitant systolic murmur.^2^

Important haemodynamic consequences of mitral valve prolapse (MVP) were shown to be usually related to some underlying condition. Marked progression of mitral regurgitation to the point of having haemodynamic significance was considered to be rare in primary BMLS.^3^

Barlow and Pocock recommended that the need for dental prophylaxis be restricted to patients in whom there was definite prolapse of a leaflet edge.^4^ Should haemodynamically significant mitral regurgitation occur, the indication for a surgical approach was in general the same as exists today and the preferred procedure was then, as now, mitral valvuloplasty. The interactions between mitral valve billow and prolapse, whether primary or secondary, were fully discussed and demonstrated by Barlow and his co-workers in 1981.^3^ This communication describes the mechanism of mitral valve billow and prolapse in a variety of conditions, including primary mitral valve billow. Furthermore, the differentiation between mitral valve billow and prolapse, as well as their relationships have been clarified by Barlow and Pocock.^5^,^6^

There has been great international interest in the primary MVB (Barlow’s syndrome). This followed the first major description in 90 patients in 1968.^7^ This publication has received at least three citations.^8^-^10^

The synthesis of the syndrome started with proof that non-ejection clicks originated at the mitral valve. These clicks were originally thought to be extra-cardiac and it was Reid who initiated that concept of an intracardiac origin.^11^ Reid, however, postulated that the click arose from the chordae. The final proof of its origin at the mitral valve was made by Barlow and his coworkers. 12,13 The association of the apical murmur in late systole with a mitral abnormality was made by these workers and therefore formed part of the hallmark of MVB.

The prevalence of the primary BML syndrome is unclear. Barlow emphasised that the existence of a syndrome is predicated on specific features that are frequently associated. These are symptoms, including chest pain, electrocardiographic changes, arrhythmias, conduction defects, systemic emboli, autonomic disorders and possibly myocardial dysfunction.^1^ Indeed, it is the protean nature of these associated features which have led to the over-diagnosis of the syndrome. Such over-diagnosis accounts for the reported incidence being as high as 18%. An unfortunate consequence of over-diagnosis has been a not-infrequent failure to recognise the underlying cause and therefore appropriate treatment for cases of secondary mitral valve billow or prolapse. This error might at times follow the recognition of the associated features without good proof of the hallmark.

It is important therefore to examine some of the features of the more important and frequently associated symptoms of the primary BMLS.

## Electrocardiographic features

Barlow stated that the prevalence of associated electrocardiographic abnormalities, as recorded in different series, must be dependant on the selection of patients.^13^,^14^ The exact prevalence is unknown. The most common pattern is T-wave inversion in leads II, III and AVF. Similar T-wave abnormalities have been reported in the anterior leads and disease may or may not be associated with changes in the inferior leads. Prominent upright U-waves were also seen to be quite common, especially in the right precordial leads. Spontaneous alterations in the ECG are well known to occur and can change very rapidly in charge. The importance of these descriptions is their differentiation from some electrocardiographic patterns encountered with ischaemic heart disease and hypertrophic cardiomyopathy. All three conditions may present with chest pain and electrocardiographic abnormalities.

## Chest pain

This is a feature of the syndrome and is usually described as being severe, sharp, frequent and localised. It is rarely suggestive of angina pectoris. However, in view of the associated ECG changes and the variable nature of anginal pain, an important task is to characterise the ECG findings, particularly in relation to obstructive coronary artery disease. Barlow did this very elegantly in 1985.^15^ The basis of the proposed analytical method was a careful analysis of the time course of effort-induced ECG changes, particularly in relation to the S-T and T waves. These observations and subsequent recommendations have been validated and found to be repeatable in clinical practice, as applied to many pathological conditions. The criteria proposed by Barlow are based on the observed effects of evolving, devolving and maximised oxygen debt as brought out by effort on the electrocardiogram. This is in contrast to more conventional electrocardiographic criteria, which are primarily empirical and epidemiologically based. It is anticipated that Barlow’s criteria will stand the test of time.

## Arrhythmias

Part of the signature of primary BMLS is the association with various arrhythmias. Ventricular ectopy, varying from mild to severe, largely according to the system of Bernard Lown were frequently present. The arrhythmias were attributed to tension within the mitral valve mechanism. The proposed therapeutic approach was always conservative, mainly propranolol. It is of great interest that we now know that a specific (idiopathic) ventricular tachycardia arises in the mitral annulus, has a typical electrocardiographic appearance, may be highly symptomatic and disabling and potentially, albeit rarely, lethal. This ventricular tachycardia can be ‘cured’ by radiofrequency ablation at the mitral annulus.^16^

The association of supra-ventricular tachycardias and billowing or prolapse of the mitral valve is far more complex. Atrial fibrillation is most often associated with progressive mitral prolapse and the development of haemodynamically significant mitral regurgitation. Barlow did not consider this to be part of the syndrome although he did point out that mitral valve billow could be an early stage of progressive and often severe prolapse, particularly of myxomatous valves. The association with atrial ectopy and sustained supra-ventricular tachycardias has not as yet been independently demonstrated, although atrial ectopy and sustained supra-ventricular tachycardias may arise from the mitral annulus. The mechanisms of the commonest supraventricular tachycardias are, however, currently well known and relate to re-entry, either utilising an accessory pathway or within the atrio-ventricular node. Therefore, it is inappropriate to attribute recurrent, sustained supra-ventricular tachycardias to Barlow’s syndrome on the basis of an associated isolated or dull non-ejection systolic click or mitral murmur. Such practice, which is regrettably fairly common, denies the sufferer the real possibility of a cure by a simple radiofrequency ablation and a greatly improved lifestyle.

## Autonomic dysfunction and syncope

Disturbances in autonomic control have long been associated with Barlow’s syndrome. Postulates of a hyperadrenergic state or catecholamine sensitivity have been made. Postural hypotension and syncope have also been described in this context. Barlow considered that whatever autonomic disturbances were associated were based on anxiety, which was often a consequence of the unexplained symptoms of chest pain, palpitations, syncope or pre-syncope. Over the last approximately 15 years, we have come to realise that the heart may act as a sensory organ and that the effect of stimulation of type C vagal receptors may well act as a trigger for neuro-circulatory syncope.^17^,^18^ This syndrome occurs very frequently in the young (up to 30% of young people are reported to have had at least one syncopal episode) and no clear association of this with mitral valve billow has as yet been made. Similarly, excessive sinus tachycardia, sometimes associated with syncope, is not infrequently seen in the postural orthostatic tachycardia syndrome, which is often associated with failure of appropriate neurological input from the lower limbs.

## Sudden death

An association with Barlow’s syndrome and sudden death was made and commented on by Pocock and her co-workers in 1984.^19^,^20^ Barlow emphasised that when MVB or MVP were associated with the long QT syndrome, it was the ionic membrane abnormalities and not the mitral valve abnormalities which caused sudden death. Today we know that many apparently normal people may suffer sudden death due to underlying long QT syndrome, catecholaminergic polymorphic ventricular tachycardia (CPVT), idiopathic ventricular fibrillation and arrhythmogenic right ventricular cardiomyopathy at a time when the diagnostic features may be subtle and therefore missed. Since the latter conditions often appear in apparently normal people, the presence of a non-ejection click could be misconstrued in its importance.

## Platelet emboli

These may relate to small cerebral emboli with stroke or possibly with myocardial infarction, migraine^21^ or even sudden death. When myocardial infarction occurred in patients with mitral valve billow and very little coronary artery disease, Barlow treated such patients with anti-platelet therapy and nifedipine.4

Because of the numerous clinical components of this syndrome, Barlow (almost always aided and sometimes guided by Wendy Pocock, now Molyneux) dissected out the relationships of mitral valve billow to a number of particular primary conditions with which it was associated. They thus brought out many essential and basic components of such primary associated conditions. They would then accurately and clearly place these clinical manifestations and their relationship to mitral valve billow.

A proof of validity is whether an observation or theory stands the test of time. Therefore the descriptions of electrophysiological mechanisms as predicted from the surface ECG by such giants as Langendorf, Pick and Schamroth have been subsequently proven by direct intracardiac recordings and stimulation. So many current cardiological practices in this era of invasive investigation have shown that many of the observations and therapeutic implications as described by Barlow and his co-workers have not only been validated but have given therapeutic direction; a tribute to the value of clinical observation.

It often takes time and distance to realise how fortunate many were to train and develop at a medical school and hospital dedicated to hard work and clinical excellence. To learn that clinical science correctly practiced is no less powerful a weapon than ‘high science’ and burgeoning technology in unveiling and clarifying cardiac disease processes and their correct treatment. The story of primary (and secondary) mitral valve billow and prolapse are a clear indication of this. John Barlow and co-workers can point with pride not only to the syndrome but to the many other understandings that have sprung therefrom.
